# Do the Asia and Oceania Federation of Obstetrics and Gynecology members' websites provide information targeting women in the context of the COVID‐19 pandemic?

**DOI:** 10.1111/jog.14377

**Published:** 2020-08-07

**Authors:** Shikino Kikuchi, Tomoko Komagata, Hiromi Obara

**Affiliations:** ^1^ Bureau of International Health Cooperation National Center for Global Health and Medicine Tokyo Japan

Dear Editor,

The COVID‐19 infection, declared a pandemic in March 2020, is affecting the health and lives of everyone and caused significant behavioral changes everywhere. There is no evidence from other coronavirus infections (i.e. SARS and MERS) that pregnant women are more susceptible to infection with coronavirus.^1^ However, the media report that pregnant women and their families are affected in various ways, including missed opportunities for examinations due to movement restrictions; being forced to change the place and/or mode of delivery due to limited resources.^2^

Women need reliable information based on science. How can a professional society contribute to providing information to women? A review of the Asia and Oceania Federation of Obstetrics and Gynecology (AOFOG) website and members' websites was conducted in April 2020.

Of those 28 AOFOG members, 19 functioning websites were found (14 from the AOFOG membership website and five from the International Federation of Gynecology and Obstetrics website). Of those 19 websites, all provided information targeting society members – Obstetrics and Gynecology (Ob/Gyn) doctors (e.g. academic meetings, opportunities for career development). However, only two websites, Japan and Australia/New Zealand, provided information targeting women directly (e.g. general information on pregnancy and childbirth, gynecological conditions such as cervical cancer and menopause).

Of those 19 websites, only nine had the dedicated section(s) for COVID‐19. Such section(s) provide information targeting their society members – Ob/Gyn doctors only. The contents include clinical guidelines, introduction of the latest articles of Ob/Gyn in relation to COVID‐19 and the statement of the society. However, COVID‐19‐related information targeting women and pregnant women directly was not found in these dedicated section(s), except Japan which includes COVID‐19 information in the website targeting women. This might be due to the society's characteristics, as the Japan Society of Obstetrics and Gynecology defines its role not only as promoting academic pursuits but also delivering messages and opinions on women's health.^3^

As COVID‐19 is new to Ob/Gyn doctors, sharing accurate and up‐to‐date information is crucial. But women also need reliable science‐based information. They should be protected from fake information^4^ which could put them at risk. To protect women's health, professional societies' websites should be a source of expert information.

In conclusion, all Ob/Gyn societies' websites should include website(s) specifically targeting women beyond Ob/Gyn professionals. Furthermore, up‐to‐date reliable information should be provided in the context of COVID‐19 for both Ob/Gyn doctors and women through their websites as one of the essential roles of professional societies.

## Disclosure

None declared.
